# Unravelling Granulomatous‐Lymphocytic Interstitial Lung Disease: A Case of Common Variable Immunodeficiency With Unusual Clinical Features and Response to Intravenous Immunoglobulin

**DOI:** 10.1002/rcr2.70102

**Published:** 2025-02-01

**Authors:** Nirosha Pragash, Jennifer Mann, Anton Antonov

**Affiliations:** ^1^ Department of Thoracic Medicine Peninsula Health Melbourne Victoria Australia; ^2^ The Institute for Breathing and Sleep Melbourne Victoria Australia; ^3^ Monash Lung Sleep Allergy and Immunology Monash Health Melbourne Victoria Australia

**Keywords:** granulomatous, inflammation, interstitial, lymphocytic, sarcoidosis

## Abstract

Granulomatous‐lymphocytic interstitial lung disease (GL‐ILD) is a rare pulmonary complication associated with common variable immunodeficiency (CVID), complicating diagnosis due to overlapping symptoms with other chronic respiratory conditions. This case involves a 33‐year‐old male with a history of sarcoidosis, presenting with recurrent sino‐pulmonary infections, mediastinal and axillary lymphadenopathy, and significant splenomegaly. Despite initial treatment with prednisolone, his symptoms persisted, and FDG‐PET imaging showed metabolic activity in the sinuses and lymph nodes. Immunological assessment revealed markedly reduced immunoglobulin levels, leading to intravenous immunoglobulin (IVIG) therapy, which resulted in substantial improvement. A critical learning point is recognising that splenomegaly is commonly associated with CVID, which can aid in distinguishing it from other conditions. This case underscores the importance of considering CVID, with or without GL‐ILD, as a differential diagnosis in patients with persistent respiratory symptoms and granulomatous lung disease, including sarcoidosis. Further research is needed to optimise treatment strategies for this rare condition.

## Graphical abstract

Granulomatous‐lymphocytic interstitial lung disease (GL‐ILD) is a rare pulmonary complication associated with common variable immunodeficiency (CVID), complicating diagnosis due to overlapping symptoms with other chronic respiratory conditions. This case details the diagnosis and treatment a 33 year old male with a history of sarcoidosis, presenting with recurrent sino‐pulmonary infections, mediastinal and axillary lymphadenopathy, and significant splenomegaly eventually diagnosed with GL‐ILD with CVID.

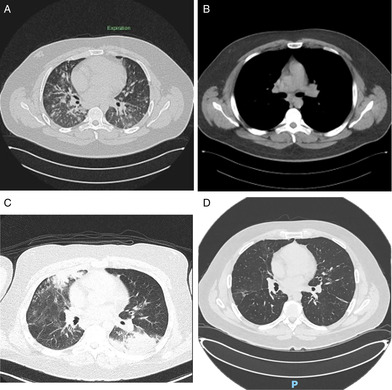

## 1 Introduction

Granulomatous‐lymphocytic interstitial lung disease (GL‐ILD) is a rare and challenging pulmonary manifestation associated with common variable immunodeficiency (CVID) [1]. GL‐ILD often presents between the ages of 20 and 50 and shows a higher prevalence in females [2]. This condition may be incidentally discovered through imaging studies, and around 15% of patients may be asymptomatic [2]. When symptoms do arise, they typically include exertional dyspnoea and cough, which can be subtle and gradually progressive.

The condition's diagnostic complexity increases due to its symptom overlap with other chronic respiratory diseases, such as sarcoidosis and chronic obstructive pulmonary disease (COPD). The presence of bronchiectasis is common in GL‐ILD; however, some patients may not exhibit this feature, complicating the diagnostic process.

In terms of prognosis, the median survival for patients with GL‐ILD can range from 5 to 10 years post‐diagnosis, depending on factors such as the underlying cause and treatment response. Lung function tests typically reveal a restrictive pattern, with reductions in forced vital capacity (FVC) and diffusing capacity of the lungs for carbon monoxide (DLCO) [3]. A decline in DLCO of more than 10% annually has been associated with poorer outcomes [3].

## 2 Case Report

**FIGURE 1:**
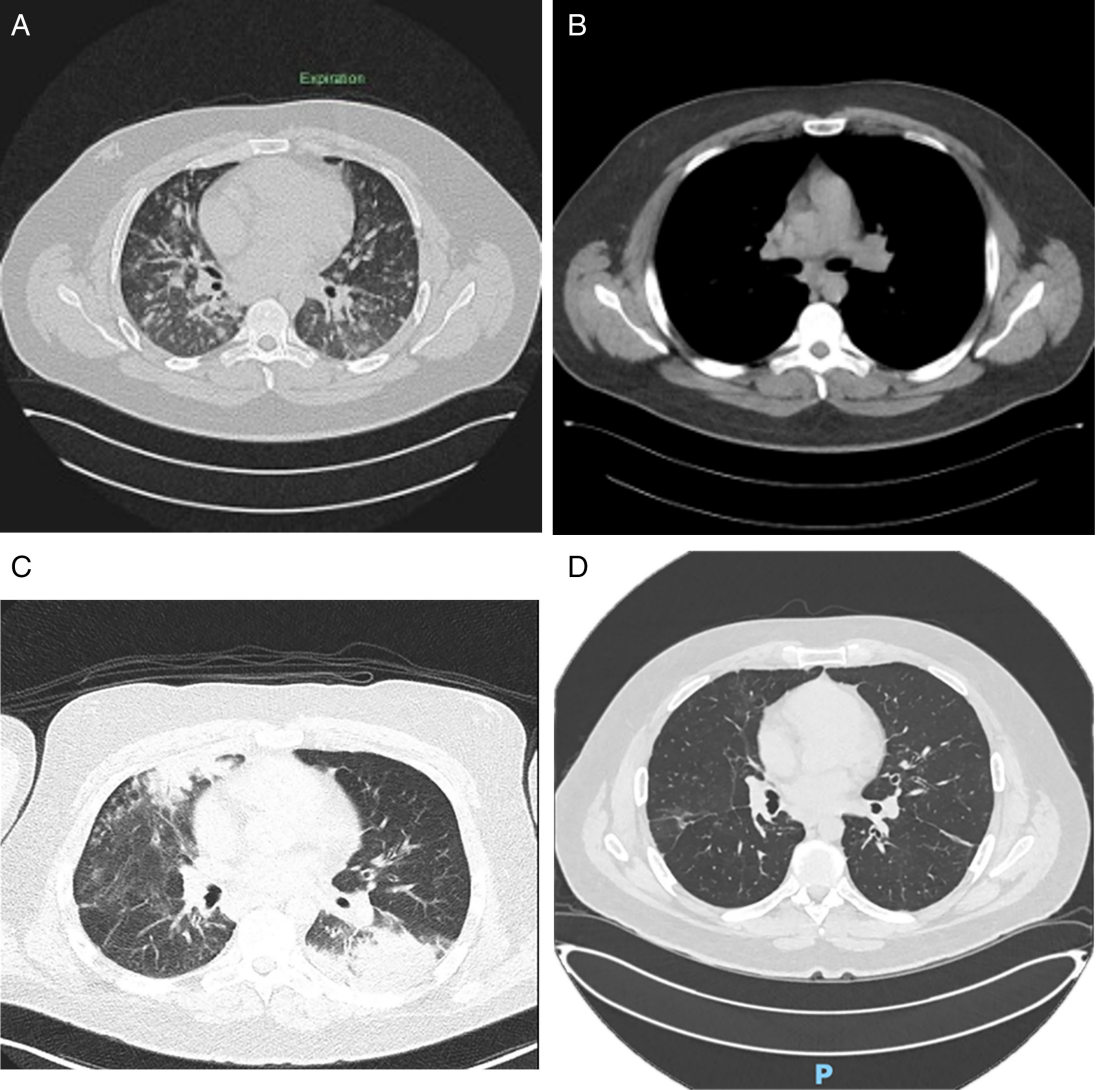
(A) CT chest (November 2017) done at the time of diagnosis demonstrating multiple peribronchovascular, subpleural and perifissural nodular ground‐glass infiltrates within the mid to lower zones. (B) CT chest (November 2017) demonstrating extensive mediastinal lymphadenopathy. (C) CT chest (September 2023) demonstrating dense consolidation left lower lobe and right upper lobe done. (D) CT chest (May 2024) demonstrating resolution of consolidation following intravenous immunoglobulin (IVIG) treatment.

A 33‐year‐old male presented with recurrent sino‐pulmonary infections against the backdrop of a suspected sarcoidosis diagnosis, which had been established based on a transbronchial lung biopsy. He was diagnosed at the age of 27 when he experienced breathlessness while playing basketball. His lung function tests revealed a preserved FEV1/FVC ratio of 95%, but a moderately impaired diffusing capacity (DLCO) of 59%. Computed tomography (CT) imaging revealed mediastinal and axillary lymphadenopathy, splenomegaly and pulmonary reticulonodular infiltrates as illustrated in Figure 1A,B. Historical bone marrow aspiration and trephine (BMAT) results were unremarkable. A bronchoscopy performed in 2021 showed mild chronic interstitial inflammation and scattered poorly formed granulomas on transbronchial biopsy.

He was started on a tapering course of prednisolone in July 2021; however, this treatment provided minimal perceived benefit. The patient also has a history of chronic rhinosinusitis (CRS) and underwent septoplasty, turbinoplasty, and functional endoscopic sinus surgery (FESS) in March 2023. Following these procedures, he was hospitalised in April 2023 with infective sinusitis and multilobar pneumonia. Ear swabs obtained during this admission were positive for Staphylococcus and Haemophilus species. Respiratory fluid cultures, including sputum microscopy, culture, and sensitivity (MCS), were obtained and found to be negative. Despite receiving antibiotics during the admission, there was no meaningful clinical improvement. This lack of response raised suspicion that the observed consolidations on chest CT might be due to inflammatory infiltrates from GL‐ILD rather than infection. Consequently, multiple courses of prednisolone were administered post‐admission to target potential inflammation, contributing to the management strategy. FDG‐PET imaging in September 2023 revealed metabolic activity in the sinuses, mediastinal and hilar lymph nodes and spleen. Azathioprine therapy was commenced as a steroid sparing agent at a low dose of 50 mg, but was discontinued after 2 weeks due to low globulin levels.

Due to persistently low total protein and globulin levels, immunoglobulin levels were assessed, revealing significantly reduced levels of immunoglobulins (IgG < 1.4 g/L, IgM < 0.21 g/L, and IgA < 0.15 g/L). The patient was subsequently managed with 40 g of intravenous immunoglobulin (IVIG) therapy monthly. Since initiating IVIG, he has not required any further courses of antibiotics. Pre‐treatment spirometry indicated an FVC of 72.2% and a DLCO of 58.1%. Following 8 months of IVIG therapy, the patient's spirometry showed marked improvement, with an FVC of 88.6% and a DLCO of 71.8%, highlighting the positive response to treatment and improvement in lung function. Additionally, repeat CT chest scan 6 months post IVIG initiation demonstrated marked resolution of airspace opacities and consolidation, as illustrated in Figure 1C,D.

Referral to an immunologist led to the diagnosis of common variable immunodeficiency (CVID) with granulomatous inflammation. The diagnosis of granulomatous—lymphocytic interstitial lung disease (GL‐ILD) was favoured over pulmonary sarcoidosis.

## 3 Discussion

GL‐ILD is a significant cause of morbidity and mortality in CVID patients, with progressive fibrosis leading to an estimated lung function decline in the DLCO by more than 10% annually and a medical survival range of 5–10 years if untreated [3]. CVID is defined by significantly reduced immunoglobulin levels, specifically low IgG and one or both of low IgA or IgM, coupled with impaired vaccine response to Tdap (Tetanus‐diphtheria‐acellular pertussis) or pneumococcal vaccination. GL‐ILD predisposes individuals to immune dysregulation and non‐infectious pulmonary manifestations [4]. Patients with CVID and GL‐ILD have a higher rate of autoimmune complications [5], which can manifest as follicular bronchiolitis, nodular lymphoid hyperplasia, granulomatous lung disease, lymphocytic interstitial pneumonia, non‐specific interstitial pneumonia, or organising pneumonia [6].

Splenomegaly can be a distinguishing feature when differentiating between sarcoidosis and CVID with GL‐ILD as summarised in Table 1. In sarcoidosis, splenomegaly is observed in approximately 5%–10% of cases, though it is often asymptomatic and may not require specific treatment unless complications such as hypersplenism arise [7]. In contrast, splenomegaly is much more common in patients with CVID, occurring in around 30%–50% of cases, and is frequently associated with autoimmune cytopenias such as autoimmune hemolytic anaemia and idiopathic thrombocytopenic purpura which is uncommon in sarcoidosis [8, 9]. This higher prevalence of splenomegaly in CVID, particularly in patients with granulomatous inflammation, serves as a useful clinical clue to differentiate it from sarcoidosis, as seen in our patient [9]. Diagnostic differentiation is essential due to the worse prognosis associated with untreated GL‐ILD, which can lead to pulmonary fibrosis and severe lung function decline. In addition to splenomegaly, features such as recurrent infections, lymphocytic interstitial pneumonia, and non‐infectious inflammation are indicative of CVID with GL‐ILD. Conversely, lupus pernio and certain cutaneous manifestations are exclusive to sarcoidosis [4].

**TABLE 1:** Key differentiating features of GL‐ILD and sarcoidosis.

Feature	GL‐ILD (CVID‐associated)	Sarcoidosis
Immunoglobulin levels	Low IgG, often with low IgA/IgM	Typically normal
Vaccine response	Poor or absent	Normal
Splenomegaly	30%–50%	5%–10%
Autoimmune cytopenias	Common	Rare
Specific skin manifestations	Absent	Lupus pernio (exclusive)
Prognosis without treatment	High risk of fibrosis and poor outcomes	Variable and better prognosis

Granulomatous lymphocytic interstitial lung disease (GL‐ILD) commonly presents between the ages of 20 and 50, with a higher incidence observed in females [2]. It is often detected incidentally through imaging studies, with approximately 15% of patients not exhibiting any symptoms [2]. When symptoms are present, they generally develop gradually, with exertional dyspnoea and cough being the most common [10]. The severity of dyspnoea typically correlates with the extent of restrictive lung disease [10]. Additionally, reduced exercise capacity, fatigue, and malaise can arise from expiratory airflow limitations, increased physiological dead space, hypoxemia, hypercapnia, or deconditioning. Persistent non‐productive cough is common, although up to 80% of patients with GL‐ILD may also experience sputum production and occasional wheezing, particularly if bronchiectasis is present [10]. The early respiratory symptoms of GL‐ILD can be challenging to diagnose due to their overlap with other chronic conditions, such as sarcoidosis and chronic obstructive pulmonary disease (COPD) [10]. When bronchiectasis is suspected, especially in individuals with primary immunodeficiency disorders, high‐resolution computed tomography (HRCT) is recommended to confirm the diagnosis.

Lung function tests usually demonstrate a restrictive pattern, characterised by decreased forced vital capacity (FVC) and reduced DLCO [3]. The most common CT chest findings in GL‐ILD include bronchiectasis (observed in over 50% of patients), which was not seen in our patient [5]. Estimates suggest that among patients with bronchiectasis related to CVID, around 25%–40% may have GL‐ILD as a coexisting condition [11]. Additional findings include small nodules without a peri‐lymphatic distribution, consolidation, ground‐glass changes, and enlarged mediastinal lymph nodes. GL‐ILD is also more commonly found in the lower lobe distribution [5].

Treatment for GL‐ILD can be challenging. There is little data to guide treatment. A survey carried out by a UK consortium of immunologists, chest physicians, radiologists, and pathologists focused on GL‐ILD highlighted gaps in treatment understanding [2]. The study revealed a shared agreement that optimising immunoglobulin therapy should occur before starting additional specific treatments for GL‐ILD [2]. Ninety percent of the participants agreed that corticosteroids should be the initial treatment, while at least 80% concurred that the second‐line options should include azathioprine, rituximab, and mycophenolate [6].

This case report details a 33‐year‐old male with a history of suspected sarcoidosis who later developed recurrent sino‐pulmonary infections. Initial diagnosis of sarcoidosis was based on imaging and biopsy findings, with persistent symptoms and radiological change prompting further investigation. Despite a treatment trial with prednisolone, the patient's condition remained problematic, leading to the diagnosis of CVID with granulomatous inflammation. Treatment with intravenous immunoglobulin (IVIG) resulted in significant clinical improvement, and further investigation suggested the possibility of GL‐ILD. A key educational takeaway from this case is the importance of considering CVID, with or without GL‐ILD, as a differential diagnosis for patients diagnosed with sarcoidosis or other granulomatous lung diseases. The presence of splenomegaly and autoimmune cytopenias, along with recurrent sino‐pulmonary infections, served as important clues in this diagnostic process. The overall clinical presentation and response to IVIG support the diagnosis of GL‐ILD.

## Author Contributions

**Nirosha Pragash:** lead in manuscript preparation and development of subsequent drafts. **Jennifer Mann:** contributed to manuscript revisions and provided critical review. **Anton Antonov:** supervised the case report and provided overall guidance.

## Ethics Statement

The authors declare that appropriate written informed consent was obtained for the publication of this manuscript and accompanying images.

## Conflicts of Interest

The authors declare no conflicts of interest.

## Data Availability

Data sharing not applicable to this article as no datasets were generated or analysed during the current study.
